# Supplementary material for: The nitrogen starvation-induced inhibitor Rts3 restrains Sit4/PP6 to gate quiescence downstream of TORC1
Source: Nat Commun. 2026 Feb 26;17:3256. doi: 10.1038/s41467-026-69693-6 (PMC13062003; doi:10.1038/s41467-026-69693-6)
Supplement: Supplementary file 1 — Supplementary Information [file 41467_2026_69693_MOESM1_ESM.pdf]

The nitrogen starvation-induced inhibitor Rts3 restrains Sit4/PP6 to gate quiescence downstream of TORC1

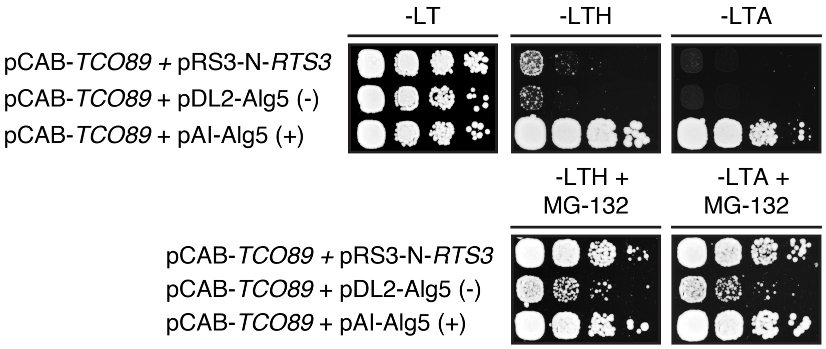

Supplementary Fig. 1 | Rts3 physically interacts with the TORC1 subunit Tco89.
Physical interaction between Tco89 and Rts3 was assessed using a membrane bound split ubiquitin yeast two-hybrid assay. The bait protein Tco89 was expressed from the pCAB vector as Cub-LexA-DBD fusion protein and the prey protein Rts3 from the pPR3-N vector as NubG-HA fusion in a strain containing the *LexAop-HIS3*, *LexAop-ADE2*, and *LexAop-LacZ* reporter genes (NMY51). pDL2-Alg5 (NubG-HA fusion) and pAI-Alg5 (NubI-HA fusion) were used as negative (-) and positive (+) control preys, respectively. Cells were spotted onto control (SD-Leu/Trp) and selective (SD-Leu/Trp/His, SD-Leu/Trp/Ade) dropout media to detect interactions. Consistent with the rapid turnover of Rts3 (see Fig. 2), a positive interaction signal was only observed when the proteasome inhibitor MG-132 (75 µM) was added to the medium to stabilize Rts3.

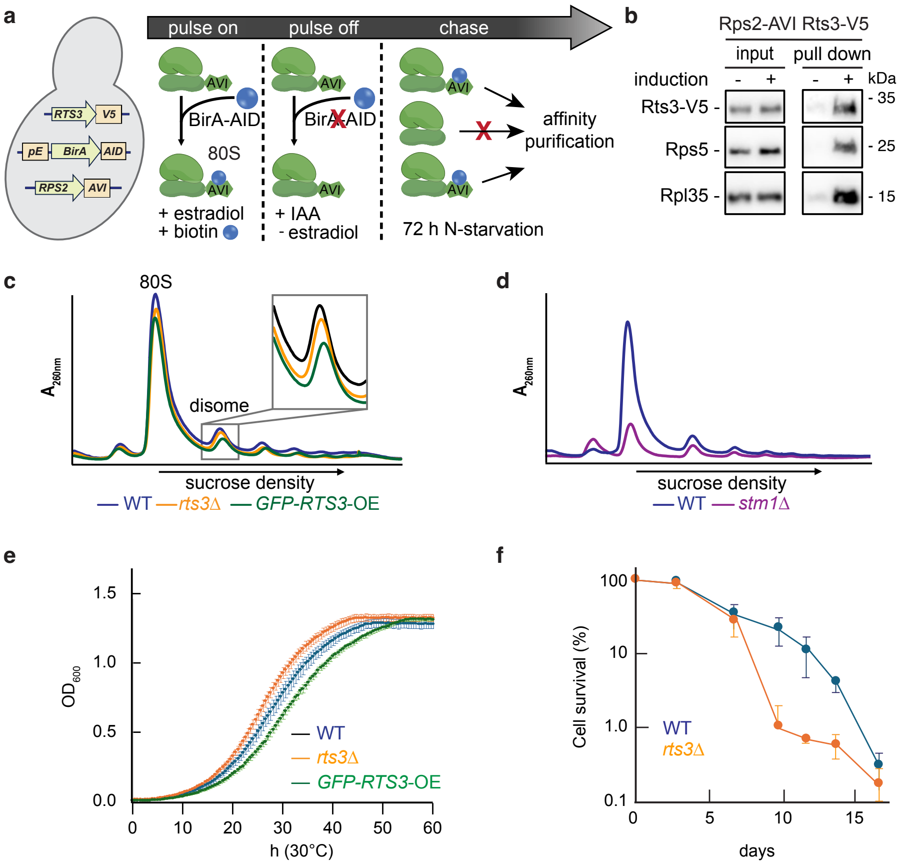

Supplementary Fig. 2 | Rts3 associates with mature ribosomes and regulates metabolic growth and chronological lifespan. a Schematic of the genotype and experimental workflow for affinity purification of proteins bound to mature ribosomes. Ribosomes were pulse-labeled by inducing a cytoplasmic BirA ligase from the estradiol-inducible promoter ("pE"; see Methods for details). Upon addition of biotin ("pulse on"), the ligase selectively biotinylated the AVI-tagged small ribosomal subunit protein Rps2. Labeling was terminated ("pulse off") by washing out estradiol and addition of indole acetic acid (+IAA) to trigger rapid BirA degradation. Cells were then nitrogen-starved for 72 h ("chase"), and biotinylated ribosomes were isolated using streptavidin-coated beads. After elution, interaction partners were verified by western blotting. **b** V5-tagged Rts3 co-eluted with biotinylated ribosomes when labeling was induced ("+") but not in control samples without biotin labeling ("-"). Total extracts (input) and affinity-purified fractions were analyzed by immunoblotting using antibodies against V5 (Rts3-V5), Rps5, or Rpl35. **c,d** Polysome profiles prepared from the indicated yeast strains were compared after 72 hours of nitrogen starvation. The position of the 80S and the disome peak (enlarged in the inset) is indicated on a representative profile. The area under the curve (AUC) of the 80S and the disome peak was quantified in 4 independent experiments and the ratio calculated (AUC_{80S}/AUC_{disome}). Only WT and *GFP-RTS3*-OE differed significantly (WT: 4.64 ± 0.15; *rts3*Δ: 4.59 ± 0.18; *GFP-RTS3*-OE: 5.18 ± 0.03; statistical significance was determined using an unpaired Student's *t*-test [WT vs. *rts3*Δ: $p = 0.6902$; WT vs. *GFP-RTS3*-OE*:* $p = 0.0005$]). Note that in contrast to *stm1*Δ, 80S hibernating ribosomes remain intact in cells with altered Rts3 levels. **e** Rts3 modulates growth on proline. Prototrophic WT, *rts3*Δ, and *GFP-RTS3*-OE strains were grown at 30°C in medium containing 0.1% proline as the sole nitrogen source. Growth was monitored by OD_{600} over time using an automated plate reader. Data are shown as mean ± SD from five independent experiments. Statistical significance was assessed by one-way ANOVA of the area under the growth curve (AUC); WT vs. *rts3*Δ, $p = 0.00268$; WT vs. *GFP-RTS3*-OE, $p = 0.00669$. **f** Chronological lifespan analysis. WT and *rts3*Δ cells were grown to stationary phase (72 h) in SDC medium and maintained in the spent medium. Survival was assessed at the indicated time points by plating on YPD agar and quantifying colony-forming units (CFUs). Data are expressed as the percentage of survival relative to Day 0 (100%). Error bars represent SD ($n = 3$).

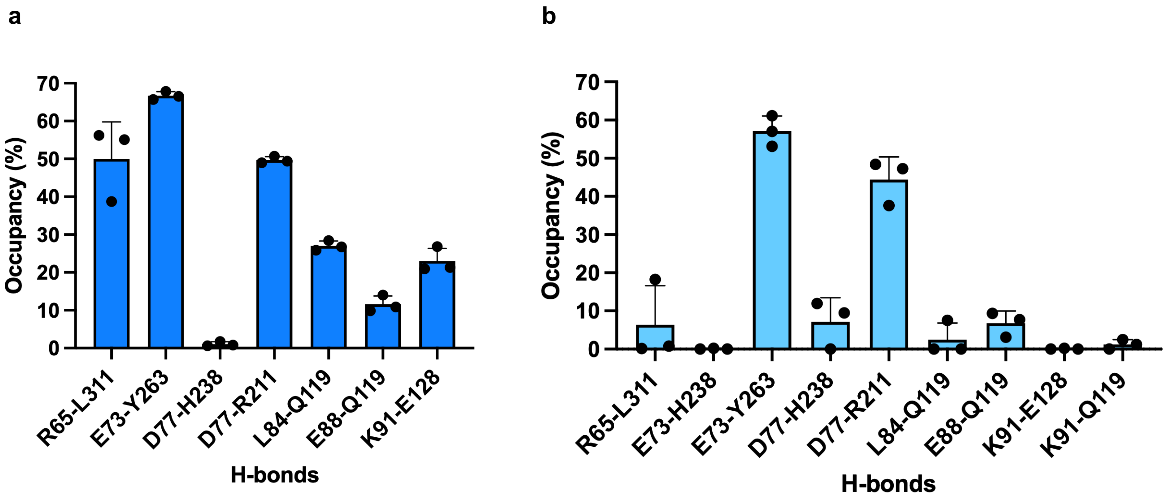

Supplementary Fig. 3 | Hydrogen bond occupancy between Rts3 and Sit4 active site residues.
a Hydrogen bond occupancy computed over the converged portion of the molecular dynamics simulation trajectory (100 to 300 ns). Residue pairs are indicated as Rts3-Sit4. **b** Hydrogen bond occupancy computed over the initial, non-converged phase of the trajectory (0 to 100 ns). Data represent the percentage of simulation frames in which a specific hydrogen bond is present. Residue pairs are indicated as Rts3-Sit4. Data (a, b) are shown as mean + SEM. The standard error of the mean was computed by dividing each trajectory fragment into three equal parts.

Supplementary Table 1 | List of antibodies, yeast strains, plasmids, and oligonucleotides

Antibodies	Source	Identifier
Mouse anti-GFP (1:3'000)	Roche	11814460001
Rabbit anti-GFP (1:4'000)	Invitrogen	PA1-980A
Rabbit anti-ADH (1:100'000)	Calbiochem	126745
Rabbit anti-ubiquitin (1:10'000)	Proteintech	80992-1-RR
Rabbit anti-Tpd3 (1:10'000)	Altmann Lab	N/A
Mouse anti-PP2A, C-subunit clone 7A6 (1:3'000)	Millipore	05-545
Rabbit anti-Sch9-pThr^{737} (1:10'000)	De Virgilio Lab	N/A
Goat anti-Sch9 (1:1'000)	De Virgilio Lab	N/A
Mouse anti-HA (16B12) (1:1'000)	ENZO	ENZ-ABS120
Goat anti-Sit4 (1:500)	Santa Cruz Biotechnology	sc-28129
Mouse anti-c-Myc (9E10) (1:3'000)	Santa Cruz Biotechnology	sc-40
Mouse anti-V5 (1:5'000)	Invitrogen	46-0705
Mouse anti-V5 (1:3'000)	BIO-RAD	MCA1360
Rabbit anti-Rps5 (1:3'000)	Panse Lab	N/A
Rabbit anti Rpl35 (1:3'000)	Panse Lab	N/A
Goat anti-mouse IgG-HRP conjugate (1:3'000)	BIO-RAD	1706516
Goat anti-rabbit IgG-HRP conjugate (1:3'000)	BIO-RAD	1706515
Rabbit anti-goat IgG-HRP conjugate (1:5'000)	BIO-RAD	1721034
anti-mouse IgG light chain, HRP conjugate (1:5'000)	Jackson ImmunoResearch	115-035-174

Yeast strains	Source	Genotype
BY4741 (Fig. 2d, 2e, 2f, 2g, 2i, S2d)	Euroscarf	*MAT a*; *his3Δ1 leu2Δ0 ura3Δ0 met15Δ0*
BY4742	Euroscarf	*MAT α*; *his3Δ1 leu2Δ0 ura3Δ0 lys2Δ0*
YL515 (Fig. 1, 2c, 5a, 5e, 5f, S2c, S2e, S2f)	Binda et al.[1]	[BY4741/2] *MAT α*; *MET15*$^{+}$ *LYS2*$^{+}$
YL516	Binda et al.[1]	[BY4741/2] *MAT a*; *MET15*$^{+}$ *LYS2*$^{+}$
KT1961 (Fig. 2b, 3a, 3b, 4j, 5g)	Pedruzzi et al.[2]	*MAT a*; *ura3-52 leu2 his3 trp1*
MP6172 (Fig. 2a, 3a, 3b)	This study	[KT1961] *URA3::RTS3p-GFP-RTS3 rts3Δ::hphNT1*
FLJ5577 (Fig. 2c)	This study	[YL515] *gat1Δ::kanMX6*
MB35 (Fig. 2c)	Binda et al.[1]	[YL515] *gln3Δ::hygR*
LD7236 (Fig. 2c)	This study	[YL515] *gln3Δ::hygR gat1Δ::kanMX6*
LD6928 (Fig. 2c, 5f)	This study	[YL516] *gcn4Δ::HIS3MX6*
MP149 (Fig. 2c)	This study	[BY4742] *rtg3Δ::kanMX6*
YSB204 (Fig. 2c)	Bontron et al.[3]	[BY4742] *msn2Δ::kanMX6 msn4Δ::kanMX6 gis1Δ::kanMX6 met15Δ0*
RL170-2C (Fig. 2h)	Loewith Lab	[TB50] *TCO89-TAP::TRP1 leu2-3 ura3-52 trp1 his3 rme1*
pup1-1 (Fig. 2d)	Li et al.[4]	[BY4741] *pup1-1*^{ts}
rpn5-1 (Fig. 2d)	Li et al.[4]	[BY4741] *rpn5-1*^{ts}
cdc4-1 (Fig. 2i)	Li et al.[4]	[BY4741] *cdc4-1*^{ts}
cdc34-1 (Fig. 2i)	Li et al.[4]	[BY4741] *cdc34-1*^{ts}
met30-9 (Fig. 2i)	Li et al.[4]	[BY4741] *met30-9*^{ts}
NP27-4A (Fig. 2i)	Lab stock	[BY4741] *grr1Δ::kanMX6*
KB22 (Fig. 3a)	This study	[KT1961] *URA3::CDC55p-GFP-CDC55 cdc55Δ::kanMX6*
MP6953 (Fig. 3a)	This study	[KT1961] *RTS1-yeGFP::HIS3MX6*

YAL3 (Fig. 3b, 5g)	This study	[KT1961] *rts3Δ::hphNT1*
MP6169 (Fig. 3b)	This study	[KT1961] *URA3::ADH1p-GFP-RTS3 rts3Δ::hphNT1*
MP4406 (Fig. 3c)	This study	[KT1961] *LST4-Envy::SpHis5*
YAL9 (Fig. 3c)	This study	[KT1961] *LST4-Envy::SpHis5 rts3Δ::hphNT1*
MP7063 (Fig. 3d)	This study	[KT1961] *URA3::RTS3p-GFP-RTS3 HIS3::CYC1p-NLS-SV40-3xmScarlet rts3Δ::hphNT1*
MP7079 (Fig. 4a)	This study	[KT1961] *URA3::RTS3p-TurboID-V5-RTS3 rts3Δ::hphNT1*
LD7381 (Fig. 4a)	This study	[KT1961] *URA3::RTS3p-TurboID-V5-Envy*
LD8593 (Fig. 4c)	This study	[BY4741] *HA-SIT4 SAP185-V5::HIS3MX6* $SAP190\text{-}myc_{13}$*::kanMX6 rts3Δ::natNT2*
LD8616 (Fig. 4c)	This study	[BY4741] *URA3::RTS3p-GFP-* $rts3^{R65A,E73A,D77A}$ *HA-SIT4 SAP185-V5::HIS3MX6* $SAP190\text{-}myc_{13}$*::kanMX6 rts3Δ::natNT2*
LD8617 (Fig. 4c)	This study	[BY4741] *URA3::RTS3p-GFP-RTS3 HA-SIT4 SAP185-V5::HIS3MX6 SAP190-* myc_{13}*::kanMX6 rts3Δ::natNT2*
YJV1275 (Fig. 4h, 4i)	Meeusen et al.[5]	[BY4741] *HA-SIT4*
LD7959 (Fig. 5g)	This study	[KT1961] *sit4Δ::kanMX6 rts3Δ::hphNT1*
LD7376 (Fig. 5g)	This study	[KT1961] *tip41Δ::kanMX6 rts3Δ::hphNT1*
LD7411 (Fig. 5g)	This study	[KT1961] *npr1Δ::kanMX6 rts3Δ::hphNT1*
LD7380 (Fig. 5g)	This study	[KT1961] *gln3Δ::kanMX6 rts3Δ::hphNT1*
MP6440 (Fig. 4j, 5g)	This study	[KT1961] *sit4Δ::kanMX6*
LD7377 (Fig. 5g)	This study	[KT1961] *tip41Δ::kanMX6*
MP5903 (Fig. 5g)	This study	[KT1961] *npr1Δ::hphNT1*
LD7188 (Fig. 5g)	This study	[KT1961] *gln3Δ::natNT2*
LD7857 (Fig. 4h, 5a, 5e)	This study	[YL515] *sit4Δ::kanMX6*
MP6406 (Fig. 5e, 5f, S2c, S2e, S2f)	This study	[YL516] *rts3Δ::hphNT1*
LD7856 (Fig. 5e)	This study	[YL516] *sit4Δ::kanMX6 rts3Δ::hphNT1*
NMY51 (Fig. S1)	Dualsystems Biotech AG	*his3Δ200 trp1-901 leu2-3,112 ade2 LYS2::(lexAop)4-HIS3 ura3::(lexAop)8- lacZ ade2::(lexAop)8-ADE2 GAL4*
yIK127 (Fig. S2a, S2b)	This study	[BY4741] *RPS2-HA-TEV-AVI pADH1-OsTir1-* myc_{9}*::HIS3 GEV-RFP::LEU2 pGAL-HA-* IAA_{2}*-BirA::URA3 RTS3-V5::hphMX4*
yIK06 (Fig. S2d)	This study	[BY4741] *stm1Δ::natMX6*

Plasmids	Source	Genes
pRS413 (Fig. 2, 3, 5, S2e, S2f)	Brachmann et al.[6]	CEN/ARS, *HIS3*
pRS414 (Fig. 2, 3)	Brachmann et al.[6]	CEN/ARS, *TRP1*
pRS415 (Fig. 2, 3, 5, S2e, S2f)	Brachmann et al.[6]	CEN/ARS, *LEU2*
pRS416 (Fig. 2, 3, 4j, 5, S2c, S2e, S2f)	Brachmann et al.[6]	CEN/ARS, *URA3*
pRS317 (Fig. 2c)	Sikorski et al.[7]	CEN/ARS, *LYS2*
YCplac33-*MET15* (Fig. 2)	Hatakeyama et al.[8]	CEN/ARS, *URA3 MET15*
pMP4008 (Fig. 2d, 2e, 2f, 2g, 2i)	This study	[pSIVh] *RTS3p-GFP-RTS3*
pMJA5093 (Fig. 2i)	This study	[pSIVh] *RTS3p-GFP-* $rts3^{S111A}$
pPW2 (Fig. 2g)	Helliwell et al.[9]	2μ, *URA3*, *TOR1-1*
pLD4540 (Fig. 2h, 4h, 4i)	This study	[pET15b] His_{6}*-RTS3*
pLD4979 (Fig. 4h, 4i)	This study	[pET15b] His_{6}*-* $rts3^{R65A,E73A,D77A}$
pLD4671 (Fig. 4j, 5e, 5f, S2c, S2e)	This study	CEN/ARS, *URA3*, *GPDp-GFP-RTS3*
pLD4976 (Fig. 4j, 5f)	This study	CEN/ARS, *URA3*, *GPDp-GFP-* $rts3^{R65A,E66A,E67A,I68A,I69A,N70A,E71A,M72A,E73A,K74A,E75A,Q76A,D77A}$

pLD4977 (Fig. 4j, 5f)	This study	CEN/ARS, *URA3*, *GPDp-GFP-rts3*^{R65A,E73A,D77A}
pLD4735 (Fig. 5a)	This study	CEN/ARS, *HIS3*, *GPDp-GFP-RTS3*
pCabWT	Dualsystems Biotech AG	CEN/ARS, *LEU2*, *Aβ-Cub-LexA-VP16*
pPR3-N	Dualsystems Biotech AG	2μ, *TRP1*, *NubG-HA*
pNP1711 (Fig. S1)	Binda et al.[1]	[pCabWT] *TCO89*
pKB4136 (Fig. S1)	This study	[pPR3-N] *RTS3*
pDL2-ALG5 (Fig. S1)	Dualsystems Biotech AG	2μ, *TRP1*, *ALG5-NubG-HA*
pAI-ALG5 (Fig. S1)	Dualsystems Biotech AG	2μ, *TRP1*, *ALG5-NubI-HA*

Oligonucleotides	Source	Sequence
YGR161C_F	Microsynth	GAAGGCTGTTGCCAAACCATGC
YGR161C_R	Microsynth	ATCACGTGCTACAGCTGGTCTC
qF-TBP1	Microsynth	CGCTACATGCCCGTAATGCAGAAT
qR-TBP1	Microsynth	TACTGGCCAGCTTTGAGTCATCCT

Supplementary References:

1. Binda, M. *et al.* The Vam6 GEF controls TORC1 by activating the EGO complex. *Mol. Cell* **35**, 563-573 (2009).
2. Pedruzzi, I. *et al.* TOR and PKA signaling pathways converge on the protein kinase Rim15 to control entry into G_0. *Mol. Cell* **12**, 1607-1613 (2003).
3. Bontron, S. *et al.* Yeast endosulfines control entry into quiescence and chronological life span by inhibiting protein phosphatase 2A. *Cell Rep.* **3**, 16-22 (2013).
4. Li, Z. *et al.* Systematic exploration of essential yeast gene function with temperature-sensitive mutants. *Nature Biotech.* **29**, 361-367 (2011).
5. Meeusen, B. *et al.* A functional map of phosphoprotein phosphatase regulation identifies an evolutionary conserved reductase for the catalytic metal ions. *bioRxiv* (2025).
6. Brachmann, C.B. *et al.* Designer deletion strains derived from *Saccharomyces cerevisiae* S288C: a useful set of strains and plasmids for PCR-mediated gene disruption and other applications. *Yeast* **14**, 115-132 (1998).
7. Sikorski, R.S. & Hieter, P. A system of shuttle vectors and yeast host strains designed for efficient manipulation of DNA in *Saccharomyces cerevisiae*. *Genetics* **122**, 19-27 (1989).
8. Hatakeyama, R. *et al.* Spatially distinct pools of TORC1 balance protein homeostasis. *Mol. Cell* **73**, 325-338 e328 (2019).
9. Helliwell, S.B. *et al.* TOR1 and TOR2 are structurally and functionally similar but not identical phosphatidylinositol kinase homologues in yeast. *Mol. Biol. Cell* **5**, 105-118 (1994).
