# Supplementary material for: The nitrogen starvation-induced inhibitor Rts3 restrains Sit4/PP6 to gate quiescence downstream of TORC1
Source: Nat Commun. 2026 Feb 26;17:3256. doi: 10.1038/s41467-026-69693-6 (PMC13062003; doi:10.1038/s41467-026-69693-6)
Supplement: Supplementary file 2 — Reporting Summary [file 41467_2026_69693_MOESM2_ESM.pdf]

nature portfolio

Corresponding author(s): Claudio De Virgilio

Last updated by author(s): Dec 2, 2025

Reporting Summary

Nature Portfolio wishes to improve the reproducibility of the work that we publish. This form provides structure for consistency and transparency in reporting. For further information on Nature Portfolio policies, see our Editorial Policies and the Editorial Policy Checklist.

Statistics

For all statistical analyses, confirm that the following items are present in the figure legend, table legend, main text, or Methods section.

n/a	Confirmed	
☐	☒	The exact sample size (*n*) for each experimental group/condition, given as a discrete number and unit of measurement
☐	☒	A statement on whether measurements were taken from distinct samples or whether the same sample was measured repeatedly
☐	☒	The statistical test(s) used AND whether they are one- or two-sided<br>*Only common tests should be described solely by name; describe more complex techniques in the Methods section.*
☒	☐	A description of all covariates tested
☐	☒	A description of any assumptions or corrections, such as tests of normality and adjustment for multiple comparisons
☐	☒	A full description of the statistical parameters including central tendency (e.g. means) or other basic estimates (e.g. regression coefficient) AND variation (e.g. standard deviation) or associated estimates of uncertainty (e.g. confidence intervals)
☐	☒	For null hypothesis testing, the test statistic (e.g. *F*, *t*, *r*) with confidence intervals, effect sizes, degrees of freedom and *P* value noted<br>*Give P values as exact values whenever suitable.*
☒	☐	For Bayesian analysis, information on the choice of priors and Markov chain Monte Carlo settings
☐	☒	For hierarchical and complex designs, identification of the appropriate level for tests and full reporting of outcomes
☒	☐	Estimates of effect sizes (e.g. Cohen's *d*, Pearson's *r*), indicating how they were calculated

Our web collection on statistics for biologists contains articles on many of the points above.

Software and code

Policy information about availability of computer code

Data collection	MS data was recorded by Xcalibur (Thermo Fisher Scientific)
Data analysis	MS data were analyzed by MaxQuant software (version 1.6.2.10), Perseus software (version 1.6.2.3), Spectronaut software version 19. Functional enrichment and interaction analyses were carried out using Cytoscape v3.10.3 with the ClueGO plugin v2.5.3, STRING database, and Omics Visualizer. Gene Ontology (GO) enrichment for the cellular compartment category was assessed relative to the S. cerevisiae genome background, applying GO-term fusion to reduce redundancy

For manuscripts utilizing custom algorithms or software that are central to the research but not yet described in published literature, software must be made available to editors and reviewers. We strongly encourage code deposition in a community repository (e.g. GitHub). See the Nature Portfolio guidelines for submitting code & software for further information.

Data

Policy information about availability of data

All manuscripts must include a data availability statement. This statement should provide the following information, where applicable:

- Accession codes, unique identifiers, or web links for publicly available datasets
- A description of any restrictions on data availability
- For clinical datasets or third party data, please ensure that the statement adheres to our policy

All datasets generated and analyzed during this study are included in the manuscript and its supplementary files. Raw mass spectrometry proteomics data have been deposited to PRIDE with accession number PXD069824. All other raw data supporting the findings of this study have been deposited to Mendeley Data

(https://data.mendeley.com/preview/pj2dkx8jw3?a=252ec8da-f7b8-4e74-be44-39b90ce1a242). No restrictions apply to data availability. This study did not involve clinical or third-party datasets.

Research involving human participants, their data, or biological material

Policy information about studies with human participants or human data. See also policy information about sex, gender (identity/presentation), and sexual orientation and race, ethnicity and racism.

Reporting on sex and gender	not applicable
Reporting on race, ethnicity, or other socially relevant groupings	not applicable
Population characteristics	not applicable
Recruitment	not applicable
Ethics oversight	not applicable

Note that full information on the approval of the study protocol must also be provided in the manuscript.

Field-specific reporting

Please select the one below that is the best fit for your research. If you are not sure, read the appropriate sections before making your selection.

[x] Life sciences [ ] Behavioural & social sciences [ ] Ecological, evolutionary & environmental sciences

For a reference copy of the document with all sections, see nature.com/documents/nr-reporting-summary-flat.pdf

Life sciences study design

All studies must disclose on these points even when the disclosure is negative.

Sample size	Confirmed – exact n is reported for each experiment (e.g., 3 or 5 biological replicates)
Data exclusions	n/a – no data were excluded from analyses
Replication	Confirmed – all experiments were performed on independent biological replicates.
Randomization	n/a – experiments were not randomized, as cultures were treated under controlled, uniform conditions.
Blinding	n/a – blinding was not performed, as outcome measurements were objective (e.g., LC-MS/MS) and not subject to observer bias.

Reporting for specific materials, systems and methods

We require information from authors about some types of materials, experimental systems and methods used in many studies. Here, indicate whether each material, system or method listed is relevant to your study. If you are not sure if a list item applies to your research, read the appropriate section before selecting a response.

Materials & experimental systems

n/a	Involved in the study	
[ ]	[x]	Antibodies
[x]	[ ]	Eukaryotic cell lines
[x]	[ ]	Palaeontology and archaeology
[x]	[ ]	Animals and other organisms
[x]	[ ]	Clinical data
[x]	[ ]	Dual use research of concern
[x]	[ ]	Plants

Methods

n/a	Involved in the study	
[x]	[ ]	ChIP-seq
[x]	[ ]	Flow cytometry
[x]	[ ]	MRI-based neuroimaging

Antibodies

Antibodies used	Described in Supplementary Table 1.
Validation	Confirmed – All primary antibodies used in this study were validated for specificity and performance in yeast by the manufacturers and/or previous publications. Validation included recognition of the correct molecular weight band on immunoblots, absence of signal in deletion mutants when available, and consistent performance across biological replicates.

Plants

Seed stocks	not applicable
Novel plant genotypes	not applicable
Authentication	not applicable
